# Do Alzheimer's Disease Risk Gene Products Actually Act in Microglia?

**DOI:** 10.3389/fnagi.2020.589196

**Published:** 2020-12-03

**Authors:** Sadayuki Hashioka, Ken Inoue, Haruo Takeshita, Masatoshi Inagaki

**Affiliations:** ^1^Department of Psychiatry, Faculty of Medicine, Shimane University, Izumo, Japan; ^2^Research and Education Faculty, Medical Sciences Cluster, Health Service Center, Kochi University, Kochi, Japan; ^3^Department of Legal Medicine, Faculty of Medicine, Shimane University, Izumo, Japan

**Keywords:** apolipoprotein E (APOE), triggering receptor expressed on myeloid cell 2 (TREM2), CD33, disease-associated microglia, monocytes, Alzheimer's disease

## Introduction

The strongest genetic risk factor for late-onset Alzheimer's disease (AD) is allele ε4 of the apolipoprotein E (*ApoE*) gene, which was discovered in 1993 (Corder et al., 1993). In around 2010, human genome-wide association studies (GWASs) of late-onset AD cases identified novel AD risk genes, the majority of which are considered to be expressed selectively or highly in microglia (Hansen et al., 2018). These include the triggering receptor expressed on myeloid cell 2 (*TREM2*) (Jonsson et al., 2013), complement receptor 1 (*CR1*) (Lambert et al., 2009), ATP-binding cassette sub-family A membrane 7 (*ABCA7*) (Hollingworth et al., 2011), and *CD33* (Hollingworth et al., 2011). It is of note that the strength of the effect of the *TREM2* variant (odds ratio of 2.36-2.71) is similar to that of ApoE ε4 (odds ratio of 3.08) (Villegas-Llerena et al., 2016). Of these genes, *ApoE, TREM2*, and *CD33* have been identified as key genes involved in the intermediate state of disease-associated microglia (DAM, also referred to as microglial neurodegenerative phenotype) by most recent single-cell RNA sequencing studies of microglia from AD-transgenic (Tg) mice (Keren-Shaul et al., 2017; Krasemann et al., 2017). Specifically, *ApoE* and *TREM2* are up-regulated while *CD33* is down-regulated during DAM activation (Keren-Shaul et al., 2017; Krasemann et al., 2017). Furthermore, the DAM signature is dependent on the TREM2-ApoE pathway in AD-Tg mice (Krasemann et al., 2017). Immunohistochemistry on human AD brain tissue has shown that even though DAM-like microglia (labeled with only one type of DAM marker) are located in close proximity to Aβ plaques, they are not found in non-plaque areas (Keren-Shaul et al., 2017; Krasemann et al., 2017). These genetic and immunohistochemistry findings imply that the functional changes of ApoE, TREM2, and CD33 caused by the variants of GWAS-identified AD risk genes, which overlap with DAM-associated genes, alter microglial functions and consequently play important roles in AD pathogenesis. Nevertheless, it should be taken into account that the protein expression of these molecules in human brains is fundamentally different from that in rodent brains.

## TREM2 Expression In Microglia

A number of animal studies have demonstrated microglial immunoreactivity for TREM2 in the rodent brain (Thrash et al., 2009; Kawabori et al., 2015). In the human brain, however, there has been controversy about the microglial expression of TREM2. Even though human microglia isolated from AD brains show up-regulated expression of TREM2 mRNA (Gosselin et al., 2017), this increase cannot be readily confirmed at the protein level. Using the same polyclonal anti-TREM2 antibody (Sigma HPA010917), two independent immunohistochemical studies on 312 postmortem cases, including 92 AD cases, have shown that human microglia do not express the TREM2 protein (Satoh et al., 2013; Fahrenhold et al., 2018). These studies have also demonstrated that the TREM2-immunoreactive cells are recruited monocytes in brain blood vessels, not microglia or perivascular macrophages (Satoh et al., 2013; Fahrenhold et al., 2018). On the other hand, a study on 33 cases, including 11 AD cases, reported TREM2-immunoreactive microglia associated with plaques; this study employed a different polyclonal anti-TREM2 antibody (R&D AF1828) (Lue et al., 2015). Although we have no convincing reason to explain this inconsistency in microglial immunoreactivity, the HPA010917 antibody appears to be more reliable than the AF1828 antibody, based on immunoreacting ability. Whereas AF1828 can recognize human recombinant TREM2 in immunoblotting, it is not able to label monocytes, macrophages, dendritic cells, or osteoclasts in human spleen and bone marrow (Satoh et al., 2013). The discrepancy in the human microglial expression of TREM2 between mRNA levels and protein levels could be attributed to the possibility that human TREM2 protein in microglia is post-translationally modified. For example, HPA010917 may not bind to a protein modified by glycosylation. Human TREM2 has been demonstrated to undergo glycosylation (Park et al., 2015), even though it is not known whether this kind of post-translational modification changes the TREM2 antigenicity. Another possibility is that the antibody recognizes human TREM2, but since the protein is soluble in humans, it is no longer detected by immunohistochemical methods. Soluble TREM2 resulting from ectodomain shedding has been detected in human cerebrospinal fluid by the enzyme-linked immunosorbent assay (Suarez-Calvet et al., 2019). The immunohistochemical finding that human TREM2-immunoreactive cells are largely restricted to blood circulation, not to the brain, implies that an altered immune response in the periphery may play a role in the development and progression of AD.

## ApoE Expression In Microglia

ApoE is believed to be derived mainly from astrocytes in the human brain (Murakami et al., 1988). In normal human brains, ApoE is predominantly expressed by astrocytes at both mRNA (Hansen et al., 2018) and protein (Murakami et al., 1988) levels. Microglial ApoE expression is induced in AD model mice (Hansen et al., 2018). However, in human AD brains, only a portion of microglia show the immunoreactivity for ApoE (Uchihara et al., 1995), while microglial expression of the *ApoE* gene is up-regulated (Mathys et al., 2019). Such inconsistency in ApoE expression by AD microglia may stem from post-translational modification of the apolipoprotein. ApoE has recently been shown to bind to human TREM2 with a specific high affinity (Atagi et al., 2015). However, the exact relationship between ApoE and microglia in the human brain remains unknown, since microglia appear not to express the TREM2 protein in the human brain, as mentioned above. In the periphery, a significant amount of circulating ApoE is produced by macrophages as well as by the liver. It has been demonstrated that human monocyte-derived macrophages from ε4/ε4 subjects secret a large amount of ApoE but lack effective cholesterol efflux (Cullen et al., 1998). The ε4/ε4 macrophages appear to facilitate the development of hypercholesterolemia and to contribute considerably to AD pathogenesis since hypercholesterolemia is a major risk factor for late-onset AD (Meng et al., 2014).

## CD33 Expression In Microglia

CD33 (also referred to as sialic acid binding immunoglobulin-like lectin-3) used to be considered a myeloid-specific immunomodulatory receptor. Today, it is known that CD33 is also expressed by microglia at both mRNA (Galatro et al., 2017; Gosselin et al., 2017) and protein (Malik et al., 2013) levels in normal human brains. In human AD brains, the number of CD33-immunoreactive microglia is increased with a positive correlation with the insoluble Aβ42 burden (Griciuc et al., 2013). CD33 overexpression associated with the *rs3865444*^*C*^ risk allele is linked to increased AD risk, while other single nucleotide polymorphisms (i.e., *rs3865444*^*A*^ and *rs12459419*^*T*^) yield non-functional CD33 protein and reduce AD risk (Bradshaw et al., 2013; Malik et al., 2013). These genetic findings suggest that *CD33* mutations cause dichotomous results. Although the protection against AD onset could be explained by adaptive loss-of-function (Siddiqui et al., 2017), it is still unknown how *CD33* mutations contribute to AD pathogenesis. As a result of the fact that *CD33* mutations also change the immunological functions of peripheral monocytes (Bradshaw et al., 2013), the *CD33* mutation-altered peripheral immune system may contribute to AD pathogenesis to some extent, as do altered microglia functions.

## Discussion

As mentioned above, *ApoE, TREM2*, and *CD33* are human GWAS-identified AD risk factors and also DAM-associated genes identified by single-cell RNA analyses on microglia from AD model mice. Based on the cellular expression pattern of the proteins, ApoE, TREM2, and CD33 in humans, the possibility cannot be excluded that functional changes of these molecules due to gene mutations have little to do with microglia in AD pathogenesis. Instead, the altered functions of ApoE, TREM2, and CD33 may have a close link to peripheral monocytes expressing these proteins and cause a dysregulated immune response, such as chronic systemic inflammation. Furthermore, such functional changes in ApoE, TREM2, and CD33 may indirectly induce the neurotoxic activation of microglia via chronic systemic inflammation, which has been shown to induce neuroinflammation associated with microglial activation (reviewed in Hashioka et al., 2019).

## Author Contributions

SH wrote the manuscript. All authors discussed and edited the manuscript, and read and approved the final manuscript.

## Conflict of Interest

The authors declare that the research was conducted in the absence of any commercial or financial relationships that could be construed as a potential conflict of interest.
